# Inflammatory Myofibroblastic Tumor of the Right Kidney Mimicking a Locally Advanced Renal Carcinoma: A Case Report

**DOI:** 10.15586/jkcvhl.v9i4.238

**Published:** 2022-12-14

**Authors:** Ali Ariafar, Faisal Ahmed, AbdolAzim Khorshidi, Simin Torabi-Nezhad, Seyed Hossein Hosseini

**Affiliations:** 1 Urology Oncology Research Center, Department of Urology, School of Medicine, Shiraz University of Medical Sciences, Shiraz, Iran;; 2 Urology Research Center, Al-Thora General Hospital, Department of Urology, School of Medicine, Ibb University, Ibb, Yemen;; 3 Department of Urology, Dena Hospital, School of Medicine, Shiraz University of Medical Sciences, Shiraz, Iran;; 4 Department of Pathology, Dena Hospital, School of Medicine, Shiraz University of Medical Sciences, Shiraz, Iran

**Keywords:** case report, inflammatory myofibroblastic tumor, kidney, surgery

## Abstract

An inflammatory myofibroblastic tumor (IMT) is a rare neoplasm with an unclear origin that can arise anywhere on the body. It contains spindle cells (myofibroblasts) with different inflammatory elements. Primary IMT of the kidney is a clinically rare disease and is difficult to differentiate from other renal malignancies. We reported a 49-year-old male who presented with right flank pain in the past year. A computed tomography scan showed a mixed density with slight heterogeneous enhancement mass in the upper pole of the right kidney, two small hypodense nodules invading the liver, and another mass in the lateral aspect of inferior vena cava. The patient underwent right radical nephrectomy and metastasectomy. IMT was confirmed by both postoperative histopathological examination and immunohistochemical assay. The patient recovered well after the operation, and no recurrence or metastasis was noted during the 12-month follow-up.

## Introduction

An inflammatory myofibroblastic tumor (IMT) of the kidney is an uncommon neoplasm (1). The prognosis of this tumor has altered over a course of time from a benign reactive process to a high malignant potential neoplasm, depending on the many reported cases that confirm frequent and persistent clonal genetic changes (1, 2).

Nodular fasciitis, fibrous histiocytoma, and desmoid or scar tissue are its main histological features (2). IMT is most prevalent in teenagers and younger adults, and the most common location is the pulmonary system (3).

IMT treatment is not very well specified and might be difficult, and surgical diagnosis is usually required (4).

There are few cases of IMT originating from the kidney in the literature (5). In addition, an appropriate diagnosis of renal ITM allows for avoiding an unnecessary surgical intervention such as radical nephrectomy. Hence, we present a 49-year-old man diagnosed with IMT in the right kidney with multiple metastases, summarize its features to improve the knowledge of this disease, and provide appropriate care to those patients.

## Case Report

A 49-year-old man was referred to the urology outpatient clinic in May 2021 with a history of right flank pain last year. The patient was a farmer and nonsmoker; with no history of malignancy, abdominal trauma, or recent urinary tract infection.

Physical examination revealed only mild right flank tenderness without palpable mass. Urine analysis showed microscopic hematuria (15–20 RB Red blood cells (RBCs)/HPF). The blood urea nitrogen, creatinine, viral markers, and liver function tests were within the normal level.

**FIGURE 1:**
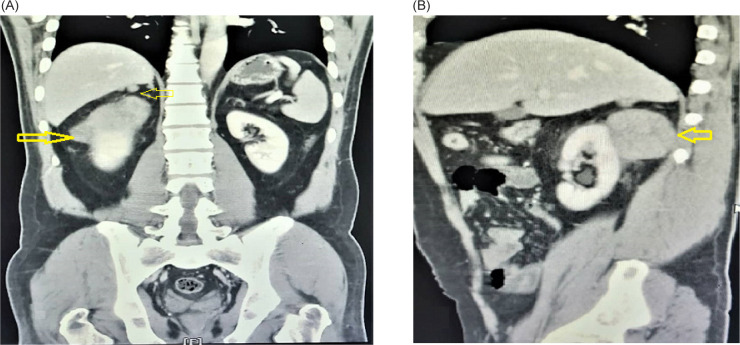
A computerized tomography (CT) scan showed: (A) Mass in the right kidney (arrow), two masses were attached to the liver tightly (arrow). (B) Oblique view of mass in the right kidney with low enhancement (arrow).

The ultrasonography (US) of the abdomen revealed an ill-defined and exophytic right renal mass, which measured 62 × 87 mm, arising from the upper pole of the right kidney. The abdominopelvic computed tomography (CT) scan revealed lobulated soft tissue mass in the posterior pararenal space, compressing the right kidney. The mass had mild contrast enhancement and cystic change without calcification and measured about 71 × 45 × 54 mm. There were two hypodense nodule masses measuring about 12 × 8 mm and 6 × 4 mm in the right posterosuperior aspect of pararenal space, which invades the liver organ. Another hypodense nodule mass measured about 20 × 15 mm located lateral to inferior vena cava (IVC). There was some fat stranding around the masses but no signs of organ invasion (Figure 1). Previous sonography-guided true-cut biopsy from the kidney mass showed spindle-cell tumor with smooth muscle differentiation, suggestive of low-grade sarcoma.

**FIGURE 2:**
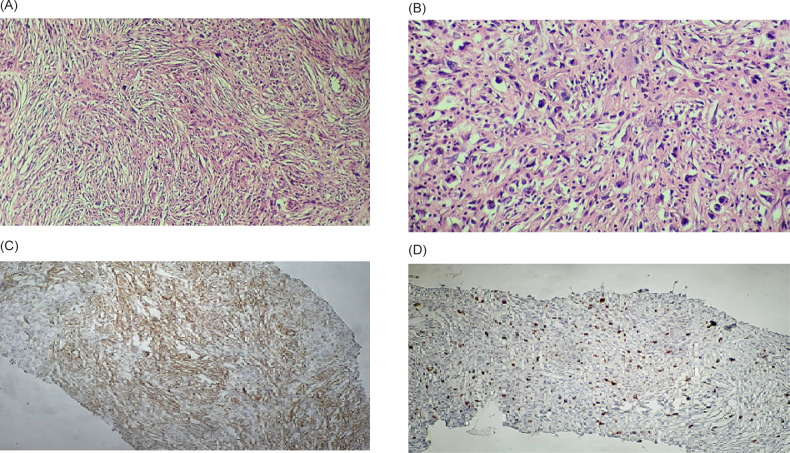
(A) Short fascicles of spindled myofibroblastic cells without overt atypia admixed with acute and chronic inflammatory cells (200×). (B) Ganglion-like polygonal cells with large rounded nuclei and prominent nucleoli (400×). Immunohistochemistry (IHC) analyses revealed positive reaction for (C) smooth muscle actin (SMA) and (D) Ki-67 (5%).

**FIGURE 3:**
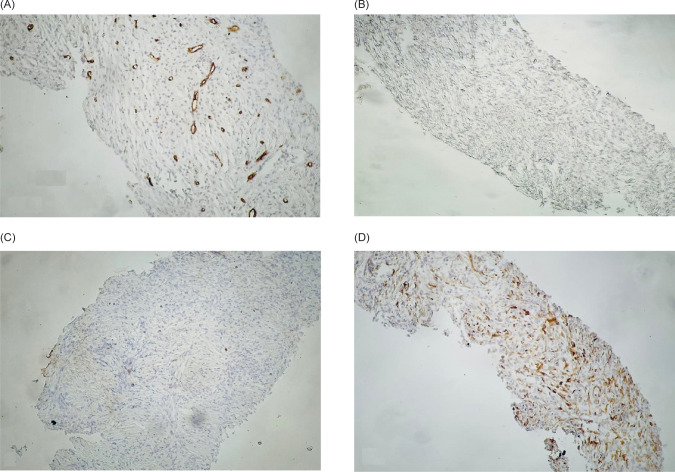
Immunohistochemistry (IHC) analyses revealed negative reactions for (A) CD34, (B) Activin receptor-like kinase (ALK), (C) Pan-cytokeratin, and (D) Desmin.

Surgical laparotomy was decided for him after a multidisciplinary discussion. Right radical nephrectomy and all solid mass resections were performed without intraoperative complications. Intraoperative findings were mass measured 71 × 45 × 54 mm and raised from the upper pole of the right kidney, two small masses measuring 12 × 8 mm and 6 × 4 mm adhered to the liver, and another mass measuring 20 × 15 mm adhered to IVC, which was successfully resected by blunt and sharp separation without complication or bleeding. On the second postoperative day, the patient was discharged from the hospital without complications. The pathologic examination of the specimens showed grayish-white, well-defined, and stiff masses. Microscopically, all resected masses, myofibroblastic-like spindle cells, were widely distributed, with various infiltrating inflammatory cells, including lymphocytes and some plasma cells. The mitotic activity was low, with only 5–10 mitoses per 10 high power fields (HPFs) (Figure 2). Immunohistochemistry (IHC) analysis was positive for smooth muscle actin (SMA) and Ki-67 (5%), and negative for CD99, CD34, activin receptor-like kinase (ALK), STAT6, Pancytokeratin, and Desmin (Figure 3).

Given the histopathological diagnosis with low-grade IMT, no additional treatment was administered. After 3 months, an enhanced CT scan revealed an empty renal lodge with no recurrence. After a 12-month follow-up, the patient was fine and had no signs of recurrence, and we planned to do an enhanced CT scan once a year for the next 5 years.

## Discussion

IMT is a rare benign condition and is thought to be an inflammatory pseudotumor rather than an actual tumoral process (6, 7). IMT is defined histologically by the growing number of typical spindle-shaped cells and inflammatory infiltration of plasma cells, eosinophils, and lymphocytes (5, 6). According to the World Health Organization classification, IMT is an intermediate biological potential tumor with a proclivity for local recurrence and a low risk of distant metastasis (6).

Regarding molecular pathways, Lu et al. reported that Upstream frameshift 1 (UPF1) mutations downregulate nonsense-mediated RNA decay (NMD), leading to NF-κB (nuclear factor kappa light chain enhancer of activated B cells) overexpression, which contributes to the immune infiltration that is characteristic of IMTs (8).

IMT is more common in children than adults without gender preference; nonetheless, extrapulmonary forms are more common in adult females, making our patient’s age and gender less prevalent (9). Although IMT has been seen in other organs, it is extremely rare in the kidney, with only a few published cases (1, 5, 7, 9).

IMT is clinically asymptomatic or may be associated with flank pain, hematuria, or hydronephrosis (1).

IMT pathogenesis and etiology remain uncertain. It could be caused by trauma, surgery-related infections, other malignant neoplasms, chronic hepatitis B infection, Epstein–Barr (EB) virus infection, or an autoimmune reaction (10). The patient in our case was a nonsmoker without a history of trauma or viral infection. There was no specific history associated with the development of IMT in our case. Similar findings were reported by Wang et al. and Abduljawad et al. (1, 7).

IMT laboratory findings were varied, with no evident abnormality in laboratory tests (1). The reported US findings of renal IMT are hypoechoic or hyperechoic change with internal vascularity. However, it may not be conclusive (7). Abdominal CT scan and magnetic resonance imaging (MRI) are helpful radiologic diagnostic methods. However, they may be associated with nonspecific findings and various clinical presentations (7). The lesions may arise from the renal cortex or the pelvis, with well-defined or ill-defined margins with varied density ranges, enhancement degrees, and patterns. In addition, cystic change and calcifications may present (11). In our case, CT revealed a mass in the posterior pararenal space arising from renal cortex; two masses invaded the liver organ and another mass was near the IVC. All masses had varied enhancement degrees, typically indicative of malignant lesions.

Due to insufficient tissue for histological examination, the Trucut biopsy may be inconclusive, and the final diagnosis is often made by histopathological examination of the surgically removed specimen (12). Similarly, the Trucut biopsy was inconclusive in our case, and the final diagnosis was made after a histopathological examination of the resected masses.

The pathologic diagnosis of this entity is challenging as well. The histology of IMT can easily be confused with sarcomas, especially myxoid leiomyosarcomas and sarcomatoid carcinomas. The best clues to its benign nature are the rarity of mitosis, edematous or myxoid stroma, a sprinkling of inflammatory cells, and prevalent erythrocyte extravasation (13). Sarcomatoid renal cell carcinoma is a high-grade tumor and is expected to have numerous mitosis and even atypical mitotic figures. IHC reveals myofibroblastic features of the IMT cells and the absence of epithelial markers in our case. ALK immunostaining can be used to confirm the diagnosis of IMT. ALK gene alterations usually accompany positive staining. However, the frequency of expression is variable, and negative ALK in tumors does not exclude the diagnosis of IMT. In addition, it is not entirely specific since ALK protein has also been detected in various sarcomas (9, 14). ALK-negative cases can be diagnosed in the presence of typical clinicopathologic findings and molecular studies (15).

The clinical significance of mitotic activity depends on the specific tumor type involved. For IMT, there is limited evidence that mitotic count and large tumor size may be associated with more aggressive clinical behavior (16).

There is no current agreement regarding managing and monitoring renal IMT. There have been no prospective studies on this subject due to the rare incidence of renal involvement (5).

Although steroid therapy has been shown to regress IMT, radical removal surgery is still considered the best treatment (1, 17). In our case, the right radical nephrectomy and resection of the other masses were performed due to multiple tightly attached lesions to the kidney, liver, and IVC.

The overall IMT recurrence rates range from 2 to 60%, with a metastatic rate of less than 5% (1, 18). However, no evidence of recurrence or metastasis was found in the renal IMTs.

IMT can occur anywhere, but it is most common in the abdominal organs, retroperitoneal space, and pulmonary system (1).

The coexistence of IMTs in the kidney, retroperitoneal space, and abdominal area could indicate a simple incidental coexistence or multiple metastases in the absence of any apparent predisposing factors and this relationship requires further investigation, as in our case (1). Similar reports of the coexistence of IMT in the kidney and other abdominal organs were reported by Wang et al. and Boualaoui et al. (1,5).

## Conclusion

Renal IMT is an uncommon tumor with unknown malignant potential. Because of the relative scarcity of renal involvement, the heterogeneity of the clinical manifestations, and the nonspecificity of the radiological signs, it is difficult to distinguish it from other types of renal malignancy. The gold standard treatment is still complete radical surgical excision.

## Consent

Written informed consent was obtained as per institutional guidelines.
